# A Phase II Multicenter Trial With Rivaroxaban in the Treatment of Livedoid Vasculopathy Assessing Pain on a Visual Analog Scale

**DOI:** 10.2196/resprot.3640

**Published:** 2014-12-10

**Authors:** Attyla Drabik, Carina Hillgruber, Tobias Goerge

**Affiliations:** ^1^Clinical Trial SupportMuensterGermany; ^2^Department of DermatologyUniversity Hospital of MuensterMuensterGermany

**Keywords:** rare disease, rivaroxaban, vasculitis, leg ulcer, skin infarction

## Abstract

**Background:**

Livedoid vasculopathy is an orphan skin disease characterized by recurrent thrombosis of the cutaneous microcirculation. It manifests itself almost exclusively in the ankles, the back of the feet, and the distal part of the lower legs. Because of the vascular occlusion, patients suffer from intense local ischemic pain. Incidence of livedoid vasculopathy is estimated to be around 1:100,000. There are currently no approved treatments for livedoid vasculopathy, making off-label therapy the only option. In Europe, thromboprophylactic treatment with low-molecular-weight heparins has become widely accepted.

**Objective:**

The aim of this trial is the statistical verification of the therapeutic effects of the anticoagulant rivaroxaban in patients suffering from livedoid vasculopathy.

**Methods:**

We performed a therapeutic phase IIa trial designed as a prospective, one-armed, multicenter, interventional series of cases with a calculated sample size of 20 patients. The primary outcome is the assessment of local pain on the visual analog scale (VAS) as an intraindividual difference of 2 values between baseline and 12 weeks.

**Results:**

Enrollment started in December 2012 and was still open at the date of submission. The study is expected to finish in November 2014.

**Conclusions:**

Livedoid vasculopathy is associated with increased thrombophilia in the cutaneous microcirculation and the continuous use of anticoagulants helps improve the symptoms. The causes of cutaneous infarctions are heterogenous, but ultimately follow the known mechanisms of the coagulation cascade. Rivaroxaban affects the coagulation cascade and inhibits the factor Xa–dependent conversion of prothrombin to thrombin, thereby considerably reducing the risk of thrombosis.

**Trial Registration:**

Trial Registration EudraCT Number: 2012-000108-13-DE; https://www.clinicaltrialsregister.eu/ctr-search/search?query=eudract_number:2012-000108-13 (Archived by WebCite at http://www.webcitation.org/6UCktWVCA); German Clinical Trials Register (DRKS): DRKS00004652; https://drks-neu.uniklinik-freiburg.de/drks_web/navigate.do?navigationId=trial.HTML&TRIAL_ID=DRKS00004652 (Archived by WebCite at http://www.webcitation.org/6UCIAKyCS).

## Introduction

### Symptomatology

Livedoid vasculopathy is an orphan skin disease characterized by recurrent thrombosis of the cutaneous microcirculation [1-6]. The disease manifests itself almost exclusively in the ankles, the back of the feet, and the distal part of the lower legs [7-9]. Symptoms above the knee joints are rarely observed. An affection of viscera by this coagulation disorder has not been reported. Livedoid vasculopathy is difficult to diagnose due to a lack of pathognomonic markers [10-15].

Recurrent thrombus formation in the cutaneous microcirculation induces local skin necrosis and ulcerations (cutaneous infarctions) leading to irreversible atrophic scars (so-called atrophie blanche). Each cutaneous infarction inevitably leaves behind residual scar tissue, causing severe and continuous cutaneous damage in the lower extremities as the disease progresses. Typical clinical symptoms of livedoid vasculopathy are acute ulcerations and white scars (atrophie blanche), both of which appear concomitantly. In addition, the impaired blood flow caused by the disease and the accompanying reduction in oxygen saturation in the dermal microcapillaries causes a visible darkening of the skin and a vascular pattern corresponding to livedo racemosa [4,16].

Due to the vascular occlusion, patients suffer intense local ischemic pain (angina cutis). Furthermore, the acute ulcerations (cutaneous infarctions) and subsequent scarring in the ankle region lead to disfigurement of the foot and a severe reduction in patients’ quality of life.

### Epidemiology

Incidence of livedoid vasculopathy is estimated at around 1:100,000 [3,6]. The disease primarily affects young women, but it has also been observed in children. However, there is a significant lack of accurate epidemiological data and related patient registers. Livedoid vasculopathy fluctuates seasonally, with an exacerbation of symptoms in summer and temporary remission in winter. It is relevant to note that the estimated incidence includes patients with livedoid vasculopathy in the context of a vasculitis [17]. Based on diagnostic data from the clinics involved, it is possible to state that incidence of the prothrombotic (noninflammatory) vasculopathy described here is in fact lower than 1:100,000.

### Pathophysiology

The histological examination of skin samples in livedoid vasculopathy cases shows occlusive fibrinoid material on the vascular walls and in the lumen of dermal microcapillaries [1,2,7,8,11]. Unlike vasculitis, inflammatory leukocyte infiltration is not normally detected [4,5,18,19]. Blood vessel occlusion occurs primarily in the microcapillaries of the middle and upper dermis, which are essential for blood supply to the epidermis. Occlusion of these blood vessels leads to insufficient perfusion and supply of the dependent skin area and triggers a strong pain signal. Impaired blood blow (ischemia) leads to so-called angina cutis, which if untreated causes a cutaneous infarction [7]. Tissue death caused by the absence of blood flow is then visible as necrosis and ulceration of the foot skin. The exact physiopathological mechanism of livedoid vasculopathy remains unclear. However, analysis in specific cases of the increased blood coagulation and thrombosis associated with the disease has enabled the detection of known prothrombotic markers that could help explain the hypercoagulability. This has made it possible to observe a relation between livedoid vasculopathy and several prothrombotic coagulation defects, such as factor V Leiden gene mutation, protein C or protein S deficiency, prothrombin G20210A gene mutation, anticardiolipin antibodies, hyperhomocysteinemia, or antithrombin III deficiency [4,6,20-25]. Alterations of the fibrinolytic system have also been detected in livedoid vasculopathy patients, including increased plasminogen activator inhibitor-1 and/or lipoprotein(a) levels [13,14]. Although the aforementioned markers have been detected in cases of systemic hypercoagulability, it remains unclear why livedoid vasculopathy only affects the lower extremities and is not detected in any organs other than the skin. Furthermore, it is not possible to detect pathological coagulation parameters in all patients suffering from the disease; therefore, it is expected that additional, hitherto undetected mechanisms causing thrombus formation may exist [17,23].

### Treatment Options

There are currently no approved treatments for livedoid vasculopathy making off-label therapy the only option. Off-label treatment of livedoid vasculopathy seeks to prevent cutaneous microvascular occlusion, yet physicians treating the disease with anticoagulants are forced to rely on case reports and treatment series [13,20,26-30]. To date, the largest number of patients who have undergone documented treatment is 9 [31]. These patients were treated with immunoglobulins, which are nonspecific and multimodal. Immunoglobulins have an anti-inflammatory and immunomodulating effect, and it is assumed that they positively influence antibody-mediated activation of the coagulation system. This form of treatment has only been carried out at a reduced number of clinics and has not become standard practice in the treatment of livedoid vasculopathy [31,32].

Although the causes of thrombus formation are extremely varied, antithrombotic treatment is generally considered a promising area in the case of livedoid vasculopathy. However, it must be applied in the ischemic phase of the disease because the necrosis that follows this critical stage inevitably leads to irreversible scarring. In Europe, thromboprophylactic treatment with low-molecular-weight heparins (eg, enoxaparin 1-2 mg/kg body weight) has become widely accepted [28,33]. These drugs have been approved for the prevention and treatment of deep vein thrombosis. Coumarins have also been described in the literature as being helpful, and the successful use of fibrinolytics, rheological drugs, and immunoglobulins have also been reported by individual clinics [20,24].

### Therapeutic Benefits of Rivaroxaban

Rivaroxaban is a novel factor Xa inhibitor used for prophylaxis of thrombosis. Treatment with the investigational medicinal product (IMP) is expected to reduce pain, prevent disfiguring after-effects, and improve the general quality of life of those treated. Experience has proven that oral administration is clearly preferable to long-term treatment with daily subcutaneous injections of heparin necessary until now. In addition, although there is also an oral version of vitamin K antagonist Marcumar available, it requires constant international normalized ratio (INR) testing [20].

At the moment, only off-label treatments exist for livedoid vasculopathy. This means that physicians have to base their recommendations on reports of individual cases when treating livedoid vasculopathy patients. This study will provide physicians treating livedoid vasculopathy with empirical evidence for choosing the right therapy and set the basis for a possible indication expansion and inclusion in reimbursement lists.

At present, there is a delay of 5 years between the first appearance of symptoms and correct diagnosis of livedoid vasculopathy [4]. The results of this study may help other health professionals recognize and treat livedoid vasculopathy at an earlier stage. In addition, publication in scientific journals and presentation at congresses will stimulate scientific debate regarding the disease and possibly serve as a starting point for other studies.

### Therapeutic Risks of Rivaroxaban

The safety of rivaroxaban was tested in 8 phase III trials with a total of 16,041 patients who received the drug by the date of protocol submission [34].

Because of its pharmacological mechanism, use of rivaroxaban may be associated with an increased risk of occult or visible bleeding in any tissue or organ, which can lead to acute posthemorrhagic anemia. Signs, symptoms, and severity (including death) vary according to location and degree of bleeding or anemia.

In clinical trials, mucosal bleeding (eg, nosebleeds, gingival, gastrointestinal, and urogenital bleeding) and anemia were observed more frequently with rivaroxaban than with vitamin K antagonist treatment [34]. Therefore, monitoring of hemoglobin/hematocrit, in addition to adequate clinical observation, may be useful in detecting occult bleeding in certain cases.

The risk of hemorrhage is higher in certain patient groups, such as those with high uncontrolled hypertension. In addition, menstrual bleeding can increase in intensity and/or duration. Some complications associated with hemorrhage are general weakness, paleness, vertigo, headache, or inexplicable swelling, as well as dyspnea and sudden shock. In some cases, an anemia can cause symptoms of cardiac ischemia, such as chest pain (angina pectoris).

Known complications of severe bleeding, such as compartment syndrome and kidney failure due to hypoperfusion, have also been reported in patients receiving rivaroxaban. Therefore, it is important that patients being treated with anticoagulants be fully aware of the increased risk of hemorrhage associated with the treatment.

Reported adverse drug reactions related to rivaroxaban have been classified according to system organ class as per the Medical Dictionary for Regulatory Activities (MedDRA) and frequency in the summary of product characteristics.

### Disadvantages of Participating in the Study

The study-related disadvantages for participants are uncertainty regarding treatment success, additional time required for extra check-ups, additional time for surveys and tests, the need to register information daily in a patient diary, and answering of questionnaires.

### Study Aim

The aim of this trial is the statistical verification of the therapeutic effects of rivaroxaban in patients suffering from livedoid vasculopathy. In addition to effectiveness, the study will also take into account quality of life, patient safety, and the use of rescue medications.

## Methods

### Trial Design

This therapeutic phase IIa (proof of concept) trial is designed as a prospective, one-armed, multicenter, interventional series of cases without a statistical interim analysis and with a calculated sample size of 20 patients (2012-000108-13-DE; DRKS00004652).

### Primary Outcome

The primary outcome is the assessment of local pain on a visual analog scale (VAS): intraindividual difference of 2 values between baseline and 12 weeks.

### Secondary Outcome

In addition to the primary study goal, we checked for the following secondary outcomes:

1. Assessment of local pain on the VAS: intraindividual difference of values between baseline and 4 weeks.
2. Assessment of local pain on the VAS: intraindividual difference of values between baseline and 8 weeks.
3. Assessment of local pain on the VAS: intraindividual difference of average values over 2 weeks between the first and the last 2 weeks of the treatment.
4. Assessment via the Dermatology Life Quality Index (DLQI): intraindividual difference of values between baseline and 4 weeks.
5. Assessment (DLQI): intraindividual difference of values between baseline and 8 weeks.
6. Assessment (DLQI): intraindividual difference of values between baseline and 12 weeks.
7. Consumption of rescue medication: averaged over the week, listed for every week of treatment.
8. Consumption of rescue medication: intraindividual difference of averaged values over 2 weeks between the first and the last 2 weeks of the treatment.

### Statistics

Statistical analysis will be performed with descriptive methods (eg, frequency tables) and statistical parameters (eg, mean, standard deviation, and quantile). Box-and-whisker plots will be created for qualitative data and bar charts for quantitative data as graphical methods. Moreover, inferential analyses will be carried out using appropriate significance tests and confidence intervals. Missing values will not be replaced.

For the primary outcome, the following 2-sided test problem is established: H0: µ=0 versus H1: µ≠0, where *μ* denotes the mean of the intraindividual difference of values of the VAS for assessing local pain between start of treatment and after 12 weeks.

The primary statistical analysis will be performed with a 2-sided exact Wilcoxon test for a significance level α=.05.

The aim of the trial is to demonstrate the therapeutic effectiveness of the study drug. Therefore, the sample size is based on this primary outcome (ie, the intraindividual difference, [“before” and “after”] in values on the VAS for assessing local pain). Therapeutic effectiveness is considered clinically relevant with a mean in the primary endpoint of at least Δ/σ=0.7. A minimum sample size of 20 evaluable patients is necessary to demonstrate a significant therapeutic effect in the primary statistical analysis with a power of 80%.

Statistical analysis of primary and secondary endpoints will be conducted according to the intention-to-treat (ITT) principle. The ITT patient population includes all patients enrolled regardless of possible protocol deviations/violations (eg, premature termination of the study or discontinuation of study medication). In addition to ITT analysis, sensitivity analyses will be conducted according to the per-protocol (PP) principle. Relevant protocol deviations leading to exclusion from the PP analysis set will be defined in the statistical analysis plan. Definition of the analysis sets will be determined in a blinded review process without knowledge of the study endpoints.

### Trial Population

It is assumed that all patients will have received some form of previous treatment for livedoid vasculopathy. For this reason, washout phases prior to the start of study treatment are defined in the inclusion and exclusion criteria.

Inclusion criteria preclude patients’ participation in other clinical studies during or within 30 days before inclusion in the present study. Study participants will be informed verbally regarding possible unforeseeable health risks and possible importation of bias into the study.

At the screening visit, patients will be asked about possible associations with the investigators or sponsor that might constitute a conflict of interest relationship of dependence (eg, relatives, employees). If a relationship of dependence is suspected, the patient cannot be included in the study.

Individuals described in § 40 Abs 4 und § 41 Abs 2 and 3 of the German drug law (Arzneimittelgesetz, AMG) are excluded from participation in the study.

Men and women will be included in this study. The expected ratio of male/female patients will be 1:3. No selection according to gender will take place for study inclusion.

### Inclusion Criteria

The following inclusion and exclusion criteria were defined:

1. Definite diagnosis of livedoid vasculopathy;
2. Age ≥18 and <80 years;
3. 40 points on the pain VAS on at least 1 of the 7 days prior to treatment start;
4. No participation in another intervention study within 30 days prior to treatment start;
5. Adequate communication skills in the German language; and
6. Patient must be able to recognize the nature, significance, and scope of the clinical trial and act accordingly.

### Exclusion Criteria

1. Known allergy to the trial medication;
2. Known problems of galactose intolerance, lactase deficiency, or glucose-galactose malabsorption;
3. Pregnancy;
4. In women: insufficiently reliable contraception methods (requirement: Pearl Index <1);
5. Lactation;
6. Known renal impairment (creatinine clearance <30 mL/min);
7. Known liver disease (Child-Pugh score B and C);
8. Known ulcerative gastrointestinal disorders within 30 days before treatment start or during treatment;
9. Uncontrolled, severe arterial hypertension (stage 3);
10. Artificial heart valves;
11. Acute pulmonary embolism;
12. Bronchiectasis or pulmonary bleeding in the patient medical history;
13. Known vascular retinopathy;
14. Intracranial or intracerebral hemorrhage within 30 days before trial start or during trial;
15. Brain, spinal cord, or eye surgery within the 30 days before trial start or during trial;
16. Spinal/epidural anesthesia or puncture within 2 weeks before treatment or during trial;
17. Administration of systemic heparin within 7 days before treatment;
18. Use of nonsteroid antirheumatic (NSAR) drugs or platelet aggregation inhibitors within 7 days before treatment or during trial;
19. Use of vitamin K antagonists (phenprocoumon, warfarin) and/or thrombin inhibitors (dabigatran) within 7 days before treatment or during trial;
20. Concomitant administration of CYP3A4 inductors (eg, rifampicin, phenytoin, carbamazepine, phenobarbital, and St. John’s wort);
21. Concomitant systemic treatment with azole antifungals (eg, ketoconazole, itraconazole, voriconazole, and posaconazole);
22. Concomitant systemic treatment with human immunodeficiency virus (HIV) protease inhibitors (eg, ritonavir); and
23. Concomitant systemic treatment with dronedarone.

### Selection of Study Centers

Study centers were selected according to their focus on the indication being studied and their clinical experience treating patients with livedoid vasculopathy. Because facilities specialized in the treatment of this disease are rare in Germany, it is expected that patients from a wider radius will attend the study centers.

### Treatment Schedule

Patients will take 1 film-coated tablet of rivaroxaban orally with food each morning and evening for 12 weeks. After 7 pain-free days, the dose will be reduced to 1 tablet each morning. If pain returns, the dose will immediately be increased to the original dose of 1 tablet each morning and evening.

### Schedule of Investigations (Visits)

A total of 4 visits are foreseen: at baseline, 2 interim visits after 4 and 8 weeks, and a final visit after 12 weeks. Deviation of 2 days is allowed. A shortening or lengthening of the interval before a visit will be compensated to maintain the 4-week examination rhythm.

Entries in patient diaries will be made on a daily basis and, if possible, at the same time each day. All entries have to refer to the past 24 hours. Documentation will start on the day of first tablet intake (start of therapy) and will end after 12 weeks.

### End of Interventional Patient Treatment

The regular interventional treatment for a single patient ends with the last visit after 12 weeks.

The patient is considered to have prematurely terminated the study if at least 1 of the following criteria is met:

1. Withdrawal of informed consent
2. Violation of inclusion or exclusion criteria during the study
3. If the investigator deems that further participation of the patient is not justifiable
4. Lack of compliance
5. Premature termination of the complete study
6. A decrease in hemoglobin level of 2 units from baseline, if bleeding cannot be stopped

Further treatment of patients with the study drug after the trial ends is not planned. The optimal treatment regimen for the patient will be discussed.

Continuous medical attendance is granted because only study centers with a focus on the treatment of livedoid vasculopathy will be selected.

### Stopping Rules

The study as a whole will end when all queries generated by the corresponding data management department are resolved and the study database is closed, but not later than 4 months after the last patient’s last visit.

In case of major contraventions of the AMG, data protection regulations, or of the principles laid down in the International Conference on Harmonisation of Technical Requirements for Registration of Pharmaceuticals for Human Use (ICH) good clinical practice guidelines (ICH E6), a study center may have to terminate the study prematurely.

Premature termination of the study as a whole will be taken into consideration if:

1. Individual protocol violations accumulate
2. Ethical or scientific justification is compromised or no longer valid
3. Serious adverse reactions occur in a large segment of the patient population, suggesting that patient safety according to the risk risk-benefit assessment can no longer be guaranteed
4. Protocol violations compromise the scientific integrity of the study with regard to planned statistical analysis or other aspects
5. Prerequisites for proper study conduct are no longer fulfilled for other reasons

Premature termination of the trial requires joint agreement between the principal investigator (sponsor) and the corresponding biostatistician. The decision has to be justified in writing.

The study may be stopped during consultations. All other study-related procedures (eg, examinations, study visits, documentation) may be continued. Therapy will be continued in a symptom-guided manner and documented according to protocol.

It has to be considered that premature termination of study treatment may lead to irrecoverable loss of patient data. The decision regarding premature termination of the study as a whole has to be jointly taken by the sponsor and the corresponding biostatistician.

### Patient Safety Monitoring

Collection and documentation of adverse events (AEs) begins when the patient signs the informed consent form and ends on the date of the last study visit for each patient (individual end of study).

All AEs have to be documented in the patient file and on the AE form of the case report forms (CRF) immediately. The investigator has to report any serious adverse event to the sponsor immediately (no longer than 24 hours after awareness).

In case of a fatal or life-threatening suspected unexpected serious adverse reaction (SUSAR), the sponsor must report relevant information immediately or within 7 days of detection to the competent ethics committee, any competent authorities and the study centers involved according to applicable legal regulations. Additional relevant information has to be provided within an additional period of 8 days.

All other cases of SUSARs that come to the attention of the sponsor must be reported immediately or within 15 days to the corresponding ethics committee, authorities, and study centers involved according to applicable legal regulations.

Annual safety update reports are to be made according to applicable legal regulations on a yearly basis or as requested, and are to be provided to the competent authorities and corresponding ethics committee.

### Documentation

Data will be recorded using CRFs. The investigator is responsible for the timely, correct, complete, and legible recording of study data in the CRFs. He/she will confirm correctness of the data by his/her signature.

CRFs are to be completed with black ballpoint pen. Corrections are to be documented as follows: the wrong entry will be crossed out with a single line and corrections will be entered next to the crossed-out text and verified by date and initials, stating the reason for the change if necessary. Instructions for use (entry and corrections) are included in each CRF.

Source data according to the ICH E6 guideline are original records of the patient file, doctor’s letters, certified copies of original records, and laboratory printouts. In addition, all patient questionnaires (self-reporting) and patient diaries are regarded as source data.

Study data are to be recorded from the patient files. As an exception, self-reporting questionnaires and patient diaries for this study will be contained in the CRF only and not in the patient files. When specifically required, data from the patient diaries will have to be transferred to the CRF.

All patient data will anonymized. Each patient will be unambiguously identified by a patient identification number assigned at the study center. The investigator will keep a patient identification list documenting the patient identification number with the patient’s full name, date of birth, sex, and date of informed consent.

The patient identification list is part of the investigator file and will remain at the study site. The patient identification number consists of a 1-digit center number, a hyphen, and a 2-digit patient number assigned for each center.

### Assurance of Data Quality

The CRFs and questionnaires will be provided with carbon paper copies. The original sheets will either be collected by the study monitor during monitoring visits and forwarded to the study coordinator, or directly requested by the study coordinator. At the study coordination center, the CRFs will be checked for completeness and consistency (in-house review). Queries will be generated for missing or implausible entries and sent to the study centers. After clarification of implausible entries and completion of missing data, CRFs and questionnaires will be handed over to the corresponding data management department.

The data management department carries out data entry with the validated study software MACRO. Data entry will be performed by 2 individuals independently. Databases resulting from first and second entries will be compared. Additional plausibility checks will be conducted regarding ranges, validity, and consistency. In case of nonplausible data, the study coordinator will be informed and he/she will forward queries to the study centers and request clarification. Answers to queries will be filed together with CRFs.

At the end of the study and after correction of all implausible data, the database will be closed. Closure of the database will be documented. The study database will be handed over to the corresponding statistics department.

### Quality Control and Quality Assurance

Quality control of the study is assured by monitoring in the study centers involved. For each monitoring visit, a monitoring report will be generated documenting the progress of the study and describing actual problems of study conduct. The explicit mode and extent of monitoring is described in a separate monitoring manual.

All investigators will declare their consent to regular visits of study monitors at the study centers. In addition, they must provide direct access to all relevant study documents including original patient documents relevant to the study.

The sponsor or designated auditors are entitled to conduct audits at the study center and other facilities participating in the study. They are entitled to inspect and review all study-relevant documents.

### Ethical and Regulatory Aspects

The study will be conducted in compliance with the current version of the Declaration of Helsinki. The present study will not begin before approval has been obtained from the leading ethics committee. The study will not be started at any additional study sites before the corresponding local ethics committee has confirmed the adequacy of the study site and the investigators.

Before inclusion in the study, the investigator will inform each patient about the nature, significance, risks, and scope of the study, as well as patients’ right to withdraw from the study at any time without prejudice. Patients will receive an informed consent form describing the study in nonscientific and generally understandable language.

The patient has to consent to study participation in writing. The investigator has to provide ample time for the patient to make a decision and allow for clarification of any questions before the consent form is signed.

According to the German drug law AMG § 40 (2a), the patients will be informed that their pseudonymized disease-related data will be stored in the study database and evaluated for scientific purposes. They have to consent to the use of their pseudonymized data in writing.

The informed consent form is to be signed by the patient and by the investigator of the study site. The patient information and consent form will be kept as 2 official (signed) copies. One copy will remain with the investigator; the other copy will be given to the patient.

This clinical study is to be carried out in conformity with the requirements of the current German drug law (AMG), all applicable legal provisions regarding data protection, and principles of good clinical practice.

The clinical study will not begin before its approval by the competent federal authority. In case of substantial amendments, a new application will be submitted. The amendment can be implemented only after approval by the competent federal authority. According to §67 AMG, the investigator must notify the local supervisory authority regarding the beginning of the study, its regular or premature termination, and any amendments. These notifications are to be made by the sponsor or an appointed designee of the sponsor on behalf of the investigator.

For the current clinical study, an insurance policy is provided and a copy of the general insurance terms and conditions will be given to the patients.

Original central study documents including CRFs have to be archived by the sponsor for at least 10 years after the end of the study. The study investigator must archive all documents of the investigator’s file and copies of the CRFs for the previously mentioned period.

After biometrical analysis, the principal investigator will generate a clinical study report (CSR). The CSR includes the clinical report, the statistical report, individual patient data listings, and conclusions. The report is to be signed by the principal investigator and by the biostatistician. Publication of the study results is the responsibility of the principal investigator irrespective of study results. A summary of the CSR has to be provided to the competent ethics committee and to the competent authority within 12 months after the end of the study. Compliance with this study protocol is mandatory. Each deviation from time schedule, scheduled study procedures, or study treatments initiated by the investigator has to be documented and justified (eg, emergency measures).

## Results

Enrollment started in December 2012 and was still open at the date of submission. The study is expected to finish in November 2014.

## Discussion

### Rationale for This Trial

Livedoid vasculopathy is associated with increased thrombophilia in the cutaneous microcirculation and the continuous use of anticoagulants helps improve the symptoms [4]. The causes of cutaneous infarctions are varied, but ultimately follow the known mechanisms of the coagulation cascade. Low-molecular-weight heparins have been shown to be effective in the treatment of livedoid vasculopathy [1,13,23].

In 2009, a new drug became available for the preventive treatment of thrombosis: the direct factor Xa inhibitor, rivaroxaban (Xarelto) [34]. Depending on the dose, rivaroxaban can have different applications. At a dose of 10 mg, prevention of venous thromboembolism in adult patients who have undergone elective hip or knee joint replacement surgery. At a dose of 15-20 mg, prevention of strokes and systemic embolism in adult patients with nonvalvular atrial fibrillation and 1 or more risk factors, such as age older than 75 years or a history of congestive heart failure, hypertension, diabetes, stroke, or transient ischemic attacks; and treatment of deep vein thrombosis (DVT) and prevention of chronic DVT and pulmonary embolism after acute DVT in adults.

Rivaroxaban affects the coagulation cascade and inhibits the factor Xa–dependent conversion of prothrombin to thrombin, thereby considerably reducing the risk of thrombosis. This mechanism has been applied successfully in isolated cases to treat livedoid vasculopathy in the skin. Therefore, it is expected that this oral treatment will help prevent cutaneous infarctions in livedoid vasculopathy patients and lead to an increase in their quality of life.

This leads to the question: How effective is rivaroxaban in treating livedoid vasculopathy?

### Risk-Benefit Analysis

The essentially prothrombotic nature of livedoid vasculopathy has been sufficiently documented and supports the use of anticoagulants in a therapeutic context [1,13,23]. The nonbinding, consensus-based application of heparin injections used until now improves the symptoms of livedoid vasculopathy, but constitutes a significant burden for patients due to the constant administration of subcutaneous injections. In addition, the effectiveness of heparin has not been demonstrated in clinical trials. The development of new oral anticoagulants is, therefore, a relevant contribution to the treatment of the disease. Treatment of individual cases has already shown the effectiveness of this therapy and participants will receive a known IMP that has already been tested on more than 16,000 patients [34]. Associated risks primarily involve a higher chance and severity of hemorrhage. However, these risks are related to anticoagulants in general and are not drug-specific. Updated indications show that—in view of safety considerations and the associated risk-benefit analysis—rivaroxaban is considered appropriate for treating severely ill patients. This includes the prevention of strokes and systemic embolism in adult patients with nonvalvular atrial fibrillation and 1 or more risk factors, such as age older than 75 years or a history of congestive heart failure, hypertension, diabetes, stroke, or transient ischemic attacks.

To increase patient safety, the trial will exclude individuals with diseases and ailments associated with a negative reaction to rivaroxaban (eg, kidney disorders, patients with increased risk of hemorrhage). In addition, administration of any concomitant medications that could produce an uncontrolled increase or decrease of rivaroxaban concentrations in plasma will not be allowed to (1) reduce the risk of hemorrhage and (2) not exceed the trial dose and mask a possible therapeutic effect. Approved treatment alternatives do not exist.

Main drawbacks for participants in the trial include the increased amount of time they will have to invest in keeping a patient diary and providing additional information (eg, surveys, questionnaires) to the research team. However, in view of the expected improvement in patient quality of life, both of these drawbacks seem acceptable.

### Discussion of the Inclusion and Exclusion Criteria

The innocuousness and effectiveness of rivaroxaban has not been proven in children aged 0-18 years and no studies for this age group exist. Therefore, only patients older than 18 years will be included in the trial.

The maximum age limit of 80 years was chosen because rivaroxaban can also be indicated for patients older than age 75 years. However, the research team must take into account that older patients, compared to younger patients, show higher plasma concentrations (mean area under the curve increase of 1.5 fold) in tests. This is primarily caused by a (apparent) reduction in total body and renal clearance. An adjustment of the dose is not considered necessary.

Because there are no data regarding the use of rivaroxaban in pregnant women, it will be necessary to ensure that reliable contraceptive measures are used. There is also a lack of data on the use of the drug during lactation. In addition, experimental animal models have indicated the transfer of rivaroxaban into breast milk. Therefore, rivaroxaban is contraindicated during breastfeeding.

The use of drugs that cause a clinically significant increase or reduction in plasma concentrations of rivaroxaban is considered an exclusion criterion for this trial.

Diseases and complaints known to have potential adverse effects on patients receiving rivaroxaban (eg, kidney malfunction, risk of hemorrhage) are also exclusion criteria for the present trial.

### Rationale for the Dosage and Treatment Duration

Depending on the dose, rivaroxaban is approved mainly for prophylactic use. The recommended dose is 15-20 mg per day. In the case of this trial, a treatment dose of 20 mg was chosen to promote more rapid pain relief in patients. This course of action is also common in the off-label use of low-molecular-weight heparins (eg, enoxaparin, 1-2 mg/kg body weight) and is widely followed in Europe because of lack of approved treatment alternatives.

Rivaroxaban has a terminal half-life of 5-13 hours and peak plasma concentration is reached after 2-4 hours [35]. To ensure the highest possible degree of therapeutic uniformity over 24 hours, the daily dose is divided into 2 doses (10 mg in the morning and 10 mg in the evening). This also seeks to prevent unnecessary peak concentrations and a higher associated risk of hemorrhage.

At 7 days after the onset of pain relief, drug intake is reduced to a maintenance dose of 10 mg per day. Considering the relationship between dose and adverse reactions, the aim is to achieve therapeutic effectiveness with the lowest possible prophylactic dose, thus further reducing the risk of adverse effects.

As described in the Introduction, livedoid vasculopathy is an incurable genetic disorder that recurs if treatment is stopped. This explains the relatively long treatment duration of 12 weeks to study the sustained therapeutic effect of the drug and document the complete regeneration and epithelialization of skin wounds over the course of the trial.

### Limitations

In the present study, pain is assessed for testing the efficacy of rivaroxaban. It would be desirable to directly measure the increased microcirculatory perfusion as an objective marker for treatment efficacy.

The calculation of the study sample size is based on assumptions found in case reports of this rare disease. The sample size was calculated for a power of 80%.

## Abbreviations

AE: adverse event
AMG: Arzneimittelgesetz (German drug law)
AR: adverse reaction
CRF: case report form
CSR: clinical study report
DLQI: Dermatology Life Quality Index
DVT: deep vein thrombosis
ICH: International Conference on Harmonisation of Technical Requirements for Registration of Pharmaceuticals for Human Use
IMP: investigational medicinal product
ITT: intention-to-treat
PP: per-protocol
SUSAR: suspected unexpected serious adverse reaction
VAS: visual analog scale
